# GLP-1 receptor agonists in neurological diseases: mechanisms and therapeutic prospects from metabolism to neuroprotection

**DOI:** 10.3389/fimmu.2026.1839620

**Published:** 2026-06-23

**Authors:** Mengting Yang, Zhigang Liang

**Affiliations:** 1 Second Clinical Medical College, Binzhou Medical University, Yantai, China; 2 Qingdao University Affiliated Yantai Yuhuangding Hospital, Yantai, China; 3 Shandong Provincial Key Laboratory of Neuroimmune Interaction and Regulation, Yantai, China; 4 National Clinical Medical Research Center for Neurological Diseases Regional Subcenter, Yantai, China

**Keywords:** blood-brain barrier, GLP-1 receptor agonists, metabolic dysfunction, neuroinflammation, neuroprotection

## Abstract

Glucagon-like peptide-1 receptor agonists (GLP-1RAs) are widely used metabolic therapies for type 2 diabetes and obesity, with well-established cardiovascular benefits. Beyond glycemic control, accumulating experimental and clinical evidence suggests that GLP-1RAs exert pleiotropic actions relevant to neurological diseases. Metabolic dysfunction, chronic inflammation, oxidative stress, mitochondrial impairment, and neurovascular injury represent convergent mechanisms that contribute to neurodegeneration, cerebrovascular pathology, and metabolism-related brain disorders. Notably, these processes overlap with pathways modulated by GLP-1 signaling across systemic and central compartments. GLP-1 receptors are expressed in neurons, glial cells, and components of the neurovascular unit, providing a biological basis for possible neurological effects. Preclinical studies suggest that GLP-1RAs can reduce neuroinflammation and oxidative stress, support mitochondrial function, and help maintain blood-brain barrier integrity. Clinical findings, however, remain inconsistent. Studies in Parkinson’s disease have reported encouraging signals, but biomarker evidence for disease modification is still limited. In Alzheimer’s disease, clinical trials have produced mixed or negative results. These differences may reflect disease stage, patient selection, drug-specific pharmacology, central nervous system exposure, endpoint sensitivity, and treatment duration. Overall, GLP-1RAs may influence neurological disease through metabolic, inflammatory, and vascular pathways, but their clinical role remains unsettled. Future studies should use biomarker-informed designs, prespecified neurological endpoints, appropriate drug selection, and sufficiently long follow-up to determine which patients and disease stages are most likely to benefit.

## 1 Introduction

Glucagon-like peptide-1 receptor agonists (GLP-1RAs) are widely used in clinical practice for the treatment of type 2 diabetes mellitus and obesity (1, 2). Beyond their established metabolic actions, robust evidence from large cardiovascular outcome trials has demonstrated that GLP-1RAs significantly reduce major adverse cardiovascular events, thereby expanding interest in their pleiotropic effects across multiple organ systems (3, 4). These findings have raised the possibility that GLP-1RAs exert clinically meaningful actions beyond glucose lowering, including potential effects on the central nervous system (5, 6).

Neurological diseases represent a leading cause of disability and mortality worldwide, yet effective disease-modifying therapies remain unavailable for many conditions, particularly neurodegenerative and cerebrovascular disorders (7). Increasing evidence indicates that metabolic dysregulation, chronic inflammation, oxidative stress, and microvascular dysfunction are central contributors to the pathogenesis of these diseases (7, 8). Notably, these pathogenic processes substantially overlap with biological pathways modulated by GLP-1RAs, providing a compelling mechanistic rationale for evaluating their therapeutic potential in neurological disorders (9, 10).

From a neurobiological perspective, the relevance of GLP-1 signaling to the nervous system is supported by the expression of glucagon-like peptide-1 receptors (GLP-1Rs) across brain regions involved in cognition, motor control, and autonomic regulation, including the hippocampus, hypothalamus, brainstem, and cerebral cortex (5, 11). Preclinical studies suggest that GLP-1RAs can attenuate neuroinflammation, reduce oxidative stress, preserve mitochondrial function, and maintain neurovascular integrity (8, 9). Nevertheless, the degree to which these effects require direct central nervous system exposure remains uncertain, and peripheral metabolic, inflammatory, endothelial, and vagal pathways may account for a substantial component of the observed neurological effects (6, 12).

The pharmacological diversity within the GLP-1RA class is clinically important. Exenatide, liraglutide, semaglutide, dulaglutide, and newer multi-receptor incretin agonists differ in molecular structure, half-life, receptor kinetics, albumin binding, and evidence for CNS exposure. Neurological effects may therefore differ among agents, and pharmacokinetic differences may partly explain inconsistent clinical findings.

**Figure 1:**
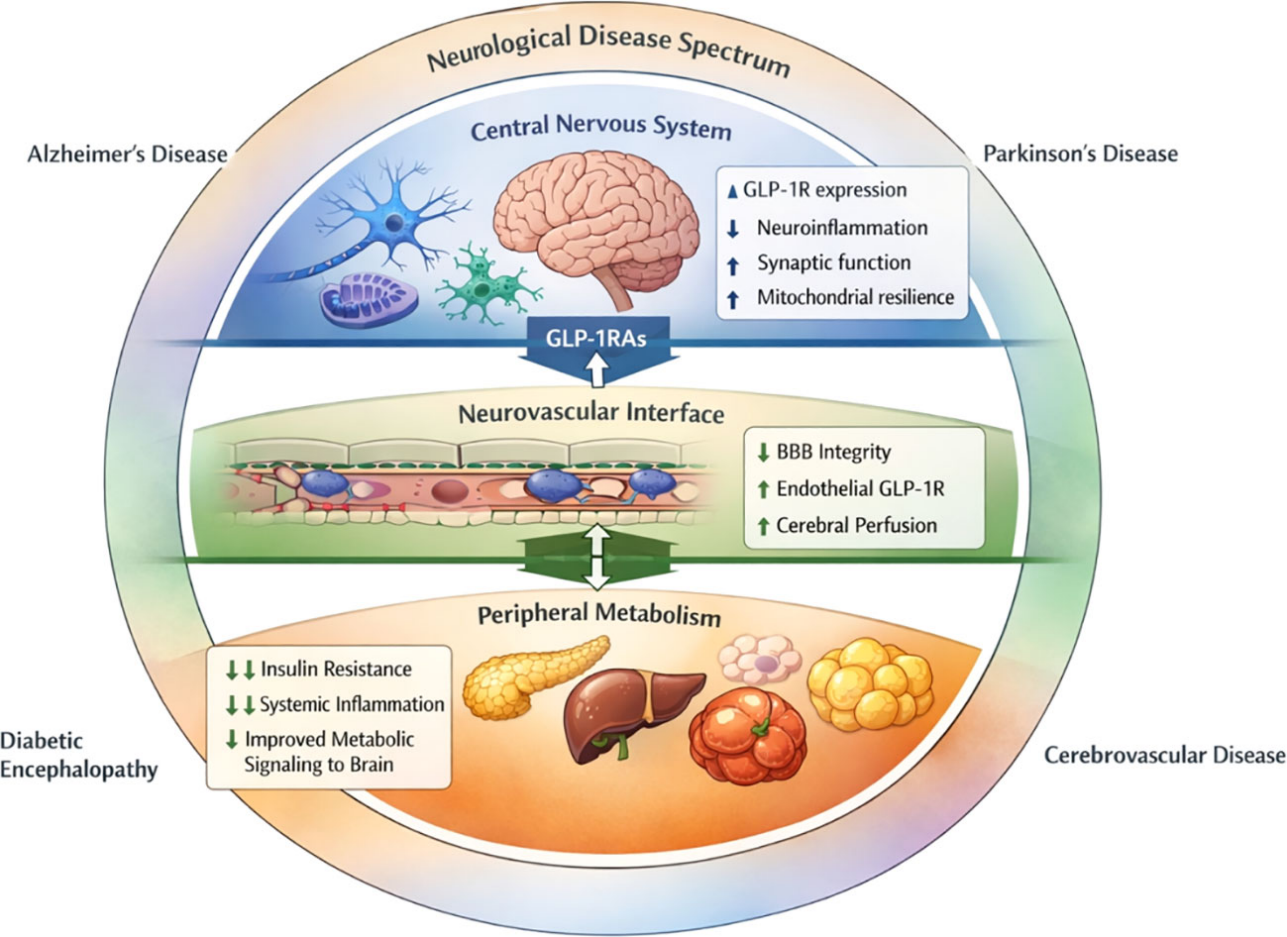
Multilevel actions of GLP-1 receptor agonists on the brain–metabolism–vascular axis. Glucagon-like peptide-1 receptor agonists (GLP-1RAs) exert coordinated effects across central, vascular, and peripheral compartments that may collectively shape neurological vulnerability. In the central nervous system, GLP-1RAs may influence neuronal and glial function, decrease neuroinflammatory signaling, and support synaptic and mitochondrial resilience. At the neurovascular interface, GLP-1R signaling may enhance endothelial GLP-1R activity, preserve blood-brain barrier integrity, and improve cerebral perfusion. In peripheral metabolic tissues, GLP-1RAs reduce insulin resistance and systemic inflammation and may improve metabolic signaling to the brain. The arrows indicate the expected direction of change for each process. The figure summarizes possible central, neurovascular, and peripheral routes by which GLP-1RAs may affect neurological disease, without implying that direct neuronal disease modification has been established.

Over the past decade, a growing body of clinical and translational research has explored the role of GLP-1RAs across a spectrum of neurological disorders. In Alzheimer’s disease and Parkinson’s disease, multiple GLP-1RAs have demonstrated neuroprotective effects in experimental models, and early-phase clinical trials have reported signals of cognitive or motor benefit (3, 8, 13). In cerebrovascular disease, GLP-1RAs have been associated with reduced infarct volume and improved neurological outcomes in preclinical studies, while observational analyses and *post hoc* data from cardiovascular trials suggest potential benefits in patients at elevated vascular risk (9, 10). In addition, in metabolism-related brain disorders—such as obesity-associated cognitive impairment and diabetic encephalopathy—GLP-1RAs may confer therapeutic advantages by improving both systemic and central metabolic homeostasis (2, 7). These multilevel actions of GLP-1RAs across central, neurovascular, and peripheral metabolic compartments are illustrated in **Figure 1**.

Despite this growing interest, several challenges currently limit the translation of GLP-1RAs into routine neurological practice. These include heterogeneity across neurological disease entities, reliance on preclinical models that incompletely reproduce human disease, limited randomized trials with prespecified neurological endpoints, uncertainties regarding dose and treatment duration, variable blood-brain barrier penetration, and outcome measures that may be insensitive to gradual multimodal effects (13, 14). Interpretation is further complicated by *post hoc* analyses, small sample sizes, incomplete biomarker confirmation, and the tendency for positive experimental findings to receive greater attention.

In this review, we summarize current clinical and translational evidence supporting the potential use of GLP-1 receptor agonists in neurological diseases. We discuss proposed mechanisms of action relevant to clinical neurology, critically evaluate positive, neutral, and negative findings from preclinical and clinical studies, and distinguish mechanistic hypotheses from conclusions supported by clinical data. Particular attention is given to the repurposing of GLP-1RAs as therapeutic agents in neurological practice and to the limitations that must be addressed before clinical translation.

## 2 Physiological basis of GLP-1 receptor signaling in the central nervous system

### 2.1 Distribution of GLP-1 receptors in the central nervous system

The relevance of glucagon-like peptide-1 receptor agonists (GLP-1RAs) to neurological diseases is supported by the broad expression of glucagon-like peptide-1 receptors (GLP-1Rs) throughout the central nervous system (15, 16). GLP-1Rs have been identified in multiple brain regions that subserve key neurological functions, including the hypothalamus, hippocampus, brainstem, basal ganglia, and cerebral cortex (16, 17). These regions are critically involved in energy homeostasis, cognition, motor control, autonomic regulation, and emotional processing—domains that are frequently impaired across a wide range of neurological disorders.

From a clinical perspective, GLP-1R expression in the hippocampus and cerebral cortex provides an anatomical framework for the reported effects of GLP-1RAs on cognitive function in preclinical models and early-stage clinical studies of Alzheimer’s disease and other dementias (18, 19). Likewise, the presence of GLP-1Rs in the basal ganglia and brainstem supports the investigation of GLP-1RAs in movement disorders such as Parkinson’s disease, in which dopaminergic neurodegeneration, neuroinflammation, and mitochondrial dysfunction play central pathogenic roles (5, 20). Expression of GLP-1Rs within hypothalamic and brainstem nuclei governing autonomic and metabolic regulation further underscores the close bidirectional relationship between systemic metabolism and central nervous system function (15, 21).

Importantly, GLP-1Rs are expressed not only in neurons but also in non-neuronal components of the central nervous system, including astrocytes, microglia, and endothelial cells of the neurovascular unit (17, 22). This broad cellular distribution suggests that GLP-1RAs may act on multiple elements of the neural microenvironment. Such multi-compartment engagement is particularly relevant in neurological diseases, where disease progression is driven by complex interactions among neurons, glial cells, and cerebral vasculature rather than by isolated neuronal dysfunction alone (22, 23).

### 2.2 Central and peripheral mechanisms of GLP-1RA action: implications for the blood–brain barrier

A central question in the neurological application of GLP-1RAs concerns whether a given agent directly accesses the brain or acts predominantly through peripheral and neurovascular signaling. Several GLP-1RAs have shown limited blood-brain barrier (BBB) penetration in experimental systems, but the magnitude, regional distribution, receptor occupancy, and clinical relevance of direct central exposure remain incompletely defined (24, 25). This uncertainty is particularly important when extrapolating high-dose animal studies or intracerebral readouts to human clinical trials. The key clinical contexts, evidence signals, translational limitations, and implications discussed in this review are summarized in **Table 1**.

**Table 1:** Summary of selected clinical evidence and translational limitations for GLP-1RAs in neurological disorders.

Clinical context	Evidence signal	Main limitation	Implication for this review
Alzheimer’s disease	Early liraglutide studies and phase 2b data suggest biological signals, whereas semaglutide EVOKE/EVOKE+ did not meet primary cognitive endpoints (64–66).	Disease stage, biomarker selection, endpoint sensitivity, and uncertainty about direct CNS target engagement.	AD remains a plausible but unproven indication.
Parkinson’s disease	Exenatide studies reported sustained motor signals, but NLY01 did not demonstrate clear efficacy in early untreated PD (72, 73, 76).	Limited biomarker confirmation makes it difficult to distinguish symptomatic benefit from disease modification.	PD remains promising, but current evidence is not definitive.
Ischemic stroke and vascular prevention	Meta-analyses of cardiovascular outcome trials suggest reduced stroke risk in metabolic populations (88, 89).	No dedicated randomized evidence establishes acute neuroprotection after stroke onset.	Claims should emphasize prevention and vascular risk modulation rather than acute treatment.
Cerebral small vessel disease and vascular cognitive impairment	Mechanistic rationale is strong because GLP-1RAs affect endothelial inflammation, metabolism, and BBB integrity.	Direct disease-specific clinical trials and validated neurovascular endpoints are sparse.	Future work should use imaging and biomarker endpoints.

Current evidence therefore supports a mixed model of action. Direct mechanisms may involve limited BBB transport or access to circumventricular structures such as the area postrema, whereas indirect mechanisms include reduced peripheral inflammation, improved endothelial function, altered adipokine and insulin signaling, and vagal afferent activation (21, 24, 26, 27). From a clinical standpoint, these indirect pathways may be especially relevant for cerebrovascular disease, cognitive impairment associated with metabolic dysfunction, and patients with systemic inflammatory or vascular risk.

Importantly, GLP-1RAs should not be treated as a homogeneous pharmacological class in neurological contexts. Agents differ in molecular size, albumin binding, half-life, receptor engagement, dosing interval, and experimental evidence for central exposure. Exendin-based compounds, liraglutide, semaglutide, and dual incretin agonists may therefore differ in their balance of direct central, neurovascular, and peripheral actions. These agent-specific properties should be considered when interpreting clinical outcomes and designing future trials.

Collectively, available evidence indicates that GLP-1RAs need not act exclusively on neurons to exert neurologically relevant effects. Instead, their coordinated actions on systemic metabolism, peripheral inflammation, endothelial biology, and neurovascular function may converge to influence brain resilience (26, 28). This multimodal mode of action also complicates causal interpretation: clinical benefit may reflect disease prevention, vascular risk modification, symptomatic modulation, or true disease modification, and these possibilities should be separated in future research.

## 3 Neuroprotective mechanisms of GLP-1 receptor agonists

Although glucagon-like peptide-1 receptor agonists (GLP-1RAs) were originally developed as metabolic therapies, a growing body of evidence indicates that they affect several processes relevant to neurological disease (22, 26). The most plausible mechanisms for clinical translation are those that connect experimental findings with human disease biology: regulation of neuroinflammation, preservation of mitochondrial and synaptic function, stabilization of the neurovascular unit, and improvement of systemic metabolic stress (29, 30). The following sections therefore prioritize mechanisms with translational relevance rather than presenting all reported pathways as equally established.

### 3.1 Anti-inflammatory effects

Chronic inflammation represents a shared pathological hallmark of many neurological disorders, including both neurodegenerative and cerebrovascular diseases (31). Within the central nervous system, sustained activation of microglia and astrocytes promotes neuronal injury through the release of pro-inflammatory cytokines, chemokines, and reactive oxygen species (32). Preclinical studies have consistently demonstrated that GLP-1RAs attenuate neuroinflammatory responses across diverse experimental models of neurological disease (22, 33).

Mechanistically, GLP-1RAs have been shown to suppress microglial activation and promote a shift toward less pro-inflammatory microglial phenotypes, accompanied by reduced expression of key inflammatory mediators such as tumor necrosis factor-α, interleukin-1β, and interleukin-6 (33, 34). Importantly, these anti-inflammatory actions extend beyond neurons to include glial cells and components of the neurovascular unit, highlighting the capacity of GLP-1RAs to modulate inflammatory signaling at multiple cellular levels (35).

From a clinical perspective, the ability of GLP-1RAs to dampen neuroinflammation is relevant because inflammation is increasingly recognized as an active contributor to disease progression rather than only a secondary epiphenomenon (31, 36). However, human evidence linking GLP-1RA-induced anti-inflammatory effects directly to neurological outcomes remains limited. *Post hoc* analyses and early-phase clinical studies suggest that patients with inflammatory, metabolic, or vascular risk profiles may derive greater benefit, but these observations require biomarker-based confirmation before firm clinical conclusions can be drawn (37, 38).

### 3.2 Reduction of oxidative stress and preservation of mitochondrial function

Oxidative stress and mitochondrial dysfunction play central roles in neuronal degeneration and impaired recovery following neurological injury (39). Neurons are particularly susceptible to oxidative damage owing to their high energy demands and limited regenerative capacity. Experimental evidence indicates that GLP-1RAs reduce the generation of reactive oxygen species and enhance mitochondrial efficiency in both neuronal and glial cells (22, 40).

Treatment with GLP-1RAs has been associated with increased mitochondrial biogenesis, stabilization of mitochondrial membrane potential, and reduced oxidative damage to cellular macromolecules (40, 41). These effects appear to arise from a combination of direct intracellular signaling and indirect improvements in systemic metabolic control (30). In the context of neurological disease, preservation of mitochondrial function may translate into enhanced neuronal survival, improved synaptic function, and greater resilience to metabolic or ischemic stress (39, 42).

Clinically, mitochondrial dysfunction has emerged as a promising therapeutic target in neurodegenerative disorders such as Parkinson’s disease and Alzheimer’s disease (42, 43). The capacity of GLP-1RAs to modulate mitochondrial pathways therefore provides a plausible mechanistic framework for the neuroprotective signals reported in early clinical studies, although validation in larger, well-powered trials remains necessary (37, 43).

### 3.3 Protection of the blood–brain barrier and the neurovascular unit

Integrity of the blood–brain barrier (BBB) and the neurovascular unit is essential for maintaining central nervous system homeostasis (44). BBB disruption and cerebral microvascular dysfunction are common features of both acute and chronic neurological conditions, including ischemic stroke, vascular cognitive impairment, and neurodegenerative diseases (23, 45). Increasing evidence suggests that GLP-1RAs exert protective effects on the neurovascular system (46).

Preclinical studies have demonstrated that GLP-1RAs reduce endothelial inflammation, improve endothelial function, and decrease vascular permeability, thereby helping to preserve BBB integrity and limit infiltration of peripheral immune cells into the brain parenchyma (35, 46). In experimental models of cerebral ischemia, GLP-1RA treatment has been associated with reduced cerebral edema and improved neurological outcomes (47).

From a translational standpoint, neurovascular protection represents a particularly attractive therapeutic mechanism, especially in patients with overlapping neurodegenerative and vascular pathology (23, 48). By targeting endothelial dysfunction and microvascular inflammation, GLP-1RAs may offer benefits that complement traditional neuroprotective strategies focused primarily on neurons (44, 48).

### 3.4 Metabolic regulation and indirect neuroprotection

Systemic metabolic dysfunction - including insulin resistance, obesity, and dyslipidemia - is increasingly recognized as a contributor to neurological disease risk and progression (49). GLP-1RAs exert well-established effects on metabolic homeostasis, including improvements in insulin sensitivity, body weight reduction, and modulation of lipid metabolism (2, 50). These systemic actions may indirectly but substantially influence brain health, especially in populations in whom vascular and metabolic comorbidities contribute to cognitive decline or recurrent cerebrovascular risk (30, 49).

Improved insulin signaling has been linked to enhanced synaptic plasticity and cognitive performance, while weight reduction and better metabolic control may mitigate vascular risk and chronic low-grade inflammation (51, 52). In metabolism-associated brain disorders, such as diabetic encephalopathy and obesity-related cognitive impairment, these indirect mechanisms may be particularly relevant (52, 53).

Clinically, the metabolic benefits of GLP-1RAs are robust and well documented, and their neurological effects are likely mediated, at least in part, through improvements in systemic metabolic status (2, 50). This dual mode of action—combining metabolic regulation with direct and indirect neuroprotective mechanisms—positions GLP-1RAs as especially promising candidates for neurological diseases characterized by metabolic comorbidity (30, 53).

Taken together, GLP-1 receptor agonists exert a constellation of neuroprotective effects that target core pathological processes across neurological diseases (26, 29). By modulating inflammation, reducing oxidative stress, preserving mitochondrial and neurovascular function, and improving systemic metabolism, GLP-1RAs may influence disease progression through multiple complementary pathways. These mechanisms provide a strong translational rationale for ongoing and future clinical studies evaluating GLP-1RAs in neurological disorders (38, 43).

## 4 GLP-1 receptor agonists in neurodegenerative diseases

Neurodegenerative diseases are characterized by progressive neuronal dysfunction and loss, driven by complex and interrelated processes including protein aggregation, neuroinflammation, metabolic dysregulation, and neurovascular impairment (54, 55). Despite substantial advances in elucidating disease mechanisms, effective disease-modifying therapies remain limited (56). Against this backdrop, increasing attention has focused on glucagon-like peptide-1 receptor agonists (GLP-1RAs) as potential modulators of convergent pathogenic pathways implicated in neurodegeneration (22, 37, 57).

### 4.1 Alzheimer’s disease

Alzheimer’s disease (AD) is a multifactorial neurodegenerative disorder classically defined by amyloid-beta (Abeta) accumulation and tau pathology, but it is increasingly recognized as a condition involving impaired brain energy metabolism, central insulin resistance, neuroinflammation, and cerebrovascular dysfunction (31, 51, 58). These features support further investigation of GLP-1RAs in AD, although they do not by themselves establish clinical efficacy (29, 57).

#### 4.1.1 Experimental evidence and mechanistic insights

In preclinical models, GLP-1RAs have been shown to influence multiple dimensions of AD-related pathology, including reductions in Aβ burden, attenuation of tau hyperphosphorylation, preservation of synaptic integrity, and suppression of neuroinflammatory responses (22, 59, 60). Beyond these canonical hallmarks, several studies indicate that GLP-1RAs improve cerebral glucose utilization and preserve neurovascular function, suggesting a broader role in stabilizing brain metabolic and vascular homeostasis (30, 35, 61). Notably, these effects cannot be fully explained by systemic glycemic control alone, supporting the view that GLP-1RAs engage disease-relevant mechanisms intrinsic to the central nervous system (22, 57).

#### 4.1.2 Clinical evidence and translational challenges

Translation of these experimental findings into consistent clinical benefit has proven challenging. Early-phase studies in mild cognitive impairment or early AD suggested preservation of cerebral glucose metabolism or slower biomarker change (62, 63). More recently, a phase 2b trial of liraglutide in mild to moderate AD reported biological signals that supported further investigation but did not remove uncertainty about clinically meaningful disease modification (64). The EVOKE and EVOKE+ phase 3 semaglutide trials were designed to test early symptomatic AD with cognitive and functional endpoints (65), but publicly reported top-line results failed to meet primary cognitive endpoints (66). These findings make it necessary to avoid presenting AD data as uniformly supportive.

The mixed AD results emphasize several unresolved issues: whether treatment must begin before substantial neurodegeneration, whether amyloid or tau biomarker status should be required, whether metabolic or vascular subgroups are more responsive, and whether conventional cognitive scales are sufficiently sensitive to capture slow multimodal effects (56, 67, 68). Negative or neutral trials do not negate the biological relevance of GLP-1 signaling, but they do indicate that GLP-1RAs should not currently be described as established disease-modifying agents for AD. At present, AD remains a biologically plausible indication, but clinical efficacy has not been established.

### 4.2 Parkinson’s disease

Parkinson’s disease (PD) is characterized by progressive degeneration of dopaminergic neurons in the substantia nigra, accompanied by widespread neuroinflammation, mitochondrial dysfunction, and alterations in brain energy metabolism (42, 69). These pathological features align closely with biological pathways modulated by GLP-1RAs, rendering PD a particularly compelling target for translational investigation (37, 43).

#### 4.2.1 Experimental evidence and mechanistic insights

Across multiple experimental models of PD, GLP-1RAs have demonstrated consistent neuroprotective effects, including preservation of dopaminergic neurons, suppression of microglial activation, and improvement of mitochondrial function (41, 70, 71). Collectively, these findings support a model in which GLP-1RAs enhance neuronal resilience and stress tolerance rather than acting as direct dopaminergic or symptomatic agents (37, 69).

#### 4.2.2 Clinical evidence and conceptual implications

Among neurodegenerative disorders, PD has yielded some of the most encouraging clinical signals for GLP-1RA therapy. Randomized controlled trials of exenatide reported motor benefits that persisted beyond the active treatment period, raising the possibility of disease-modifying effects (72, 73). However, the interpretation of these findings remains cautious because sustained clinical differences do not by themselves prove slowed neurodegeneration. Biomarker confirmation of dopaminergic preservation, target engagement, or downstream neuroinflammatory modulation is still limited (74, 75).

The broader PD evidence base is also heterogeneous. NLY01, a pegylated exenatide analogue, did not show clear clinical efficacy in a randomized trial of early untreated PD, despite a plausible mechanistic basis (76). This result underscores the importance of agent selection, disease stage, dosing strategy, trial duration, and endpoint choice. It also shows that positive preclinical findings may not translate into measurable clinical benefit when human disease heterogeneity and slow progression are not adequately addressed.

Taken together, studies in AD and PD show that GLP-1RAs have therapeutic potential but also clear limitations (57, 67). Experimental data support effects on several disease-related mechanisms, but clinical translation has been mixed and context dependent (56, 66, 68, 76). Future studies should account for disease stage, metabolic status, agent-specific pharmacology, and biomarker evidence before drawing conclusions about disease modification.

## 5 GLP-1 receptor agonists in cerebrovascular diseases

Cerebrovascular diseases encompass a spectrum of acute and chronic conditions characterized by excitotoxicity, oxidative stress, inflammation, and microvascular dysfunction (44, 77). While reperfusion-based therapies have transformed the acute management of ischemic stroke, effective strategies to limit secondary injury, preserve neurovascular integrity, and promote long-term recovery remain limited (78, 79). These unmet needs have prompted interest in glucagon-like peptide-1 receptor agonists (GLP-1RAs) as potential modulators of post-ischemic and chronic cerebrovascular pathology (46, 47, 80).

### 5.1 Ischemic stroke

Ischemic stroke triggers a complex cascade of pathophysiological events that extends beyond the initial vascular occlusion, including excitotoxic neuronal injury, mitochondrial dysfunction, oxidative stress, inflammation, and disruption of the neurovascular unit (79, 81).

#### 5.1.1 Experimental evidence and mechanistic insights

In preclinical models of ischemic stroke, GLP-1RAs have consistently been shown to reduce infarct volume, attenuate neuroinflammatory responses, and improve neurological outcomes (46, 47, 82). These effects are frequently accompanied by preservation of endothelial function, reduction of oxidative stress, and stabilization of mitochondrial activity in both neurons and glial cells (40, 41, 83). Notably, several studies indicate that GLP-1RAs help maintain blood–brain barrier integrity, thereby limiting cerebral edema and secondary infiltration of peripheral immune cells (35, 46, 84).

Importantly, the neuroprotective actions of GLP-1RAs in experimental stroke models appear to involve coordinated effects on neuronal and vascular compartments, reinforcing the concept of the neurovascular unit as a primary therapeutic target (44, 85). This aligns with the broader recognition that successful post-stroke interventions must address integrated dysfunction across neurons, glia, and cerebral microvessels rather than focusing on a single cellular component (79, 85).

#### 5.1.2 Clinical evidence and translational considerations

Clinical evidence supporting the use of GLP-1RAs as acute ischemic stroke treatments remains indirect. Cardiovascular outcome trials and meta-analyses in type 2 diabetes or high vascular-risk populations suggest that GLP-1RAs reduce overall stroke risk, supporting a preventive vascular effect (50, 86–89). However, these data should not be interpreted as proof of acute neuroprotection after stroke onset, because most trials were not designed around acute neurological endpoints, infarct evolution, reperfusion status, or post-stroke functional recovery.

The absence of randomized clinical trials specifically testing GLP-1RAs in acute or subacute ischemic stroke highlights several translational challenges, including treatment timing, interaction with reperfusion therapy, patient stratification, endpoint selection, and safety in patients with dysphagia, autonomic instability, or frailty (78, 80, 90). Whether GLP-1RAs can meaningfully influence post-stroke recovery beyond vascular prevention therefore remains an open question.

### 5.2 Cerebral small vessel disease and vascular cognitive impairment

Cerebral small vessel disease (CSVD) is a major contributor to vascular cognitive impairment and mixed dementia and is characterized by chronic cerebral hypoperfusion, endothelial dysfunction, and progressive disruption of the blood–brain barrier (23, 91). These pathological features overlap substantially with metabolic, inflammatory, and vascular pathways regulated by GLP-1 signaling (49, 92).

#### 5.2.1 Experimental and conceptual evidence

Experimental studies suggest that GLP-1RAs improve endothelial function, reduce microvascular inflammation, and enhance cerebral perfusion in models of chronic vascular injury (35, 46, 93). By stabilizing the blood–brain barrier and modulating neuroinflammatory responses, GLP-1RAs may attenuate the progressive accumulation of white matter damage and neuronal dysfunction that underlies cognitive decline in CSVD (91, 94).

From a conceptual standpoint, CSVD represents a condition in which indirect neuroprotective mechanisms—mediated primarily through vascular and metabolic pathways—may be particularly relevant (49, 95). Rather than directly targeting neuronal loss, GLP-1RAs may influence disease trajectory by preserving microvascular integrity and mitigating chronic low-grade inflammation (92, 95).

#### 5.2.2 Clinical implications and knowledge gaps

Clinical data directly evaluating GLP-1RAs in CSVD or vascular cognitive impairment are currently sparse. Nevertheless, given the strong epidemiological links between diabetes, obesity, and small vessel pathology, GLP-1RAs may be especially relevant in patient populations with prominent metabolic comorbidity (49, 86, 96). Major barriers to clinical translation include the absence of disease-specific biomarkers and the lack of standardized cognitive and neurovascular endpoints (91, 97).

Future studies incorporating advanced neuroimaging markers of microvascular function and blood–brain barrier integrity will likely be essential for determining whether GLP-1RAs can meaningfully modify the course of CSVD-related cognitive decline (23, 94, 97).

## 6 Conceptual summary

Cerebrovascular diseases underscore the importance of targeting the neurovascular interface rather than neurons in isolation (44, 85). Evidence from experimental models suggests that GLP-1 receptor agonists modulate key processes involved in both acute ischemic injury and chronic microvascular dysfunction, including endothelial inflammation, oxidative stress, and blood–brain barrier disruption (35, 46, 82). However, clinical translation remains at an early stage, with most human data derived from indirect or observational studies (80, 86).

Collectively, these observations suggest that GLP-1RAs may be more relevant to vascular prevention and metabolic risk modification than to acute neuroprotection in cerebrovascular disease (49, 88–90, 95). Further studies should include biomarkers and outcome measures that capture neurovascular health, recurrent event risk, and cognitive trajectories rather than relying only on short-term neurological deficit scores (78, 91, 97).

## 7 GLP-1 receptor agonists in metabolism-related brain disorders

Metabolic dysfunction and neurological disease are increasingly viewed as interdependent rather than parallel processes (51, 96, 98). Beyond their established role in systemic glucose regulation, GLP-1 receptor agonists (GLP-1RAs) have emerged as modulators of the bidirectional brain–metabolism axis, linking peripheral metabolic status to central nervous system structure and function (29, 30, 99).

### 7.1 Obesity-associated cognitive decline

Obesity is associated with chronic low-grade inflammation, insulin resistance, and altered adipokine signaling, all of which contribute to cognitive impairment independent of overt diabetes (49, 52, 100). Epidemiological studies consistently demonstrate that midlife obesity increases the risk of later-life cognitive decline and dementia, indicating that metabolic stress exerts sustained effects on brain health (49, 101).

Preclinical studies show that GLP-1RAs ameliorate obesity-associated cognitive deficits through combined central and peripheral mechanisms. In models of diet-induced obesity, GLP-1RA treatment improves learning and memory performance, accompanied by reduced neuroinflammation in the hippocampus and prefrontal cortex, decreased microglial activation, and restoration of synaptic plasticity markers (102, 103).

Notably, weight loss alone does not fully account for these neurocognitive benefits. Central GLP-1 receptor signaling appears to directly influence neuronal energy homeostasis, synaptic transmission, and neuroinflammatory responses (22, 104). These findings support the view that GLP-1RAs exert intrinsic neuroprotective effects in obesity, rather than acting solely as indirect metabolic correctors (99, 105).

### 7.2 Diabetic encephalopathy and cognitive dysfunction in diabetes

Diabetes mellitus is increasingly recognized as a risk factor for cognitive impairment, often termed diabetic encephalopathy (96, 106). Chronic hyperglycemia, insulin resistance, oxidative stress, and microvascular dysfunction collectively disrupt neuronal integrity and synaptic function, particularly in memory-related brain regions (51, 107).

In experimental models, GLP-1RAs improve cognitive performance, reduce neuronal apoptosis, and attenuate oxidative stress in the diabetic brain (40, 41, 108). These effects are mediated, at least in part, by enhanced neuronal insulin signaling, improved mitochondrial function, and preservation of blood–brain barrier integrity (35, 51, 109).

The expression of GLP-1 receptors in key cognitive regions suggests that central GLP-1 signaling directly counteracts diabetes-related neuronal dysfunction (22, 104). This distinguishes GLP-1RAs from conventional glucose-lowering therapies and highlights their potential as disease-modifying agents for diabetes-associated cognitive decline (96, 110).

### 7.3 The brain–metabolism axis as a therapeutic framework

Collectively, evidence from obesity- and diabetes-related brain disorders supports a unifying framework in which GLP-1RAs modulate the brain–metabolism axis at multiple levels (29, 30, 99). Peripheral metabolic improvement, including reduced insulin resistance and systemic inflammation, converges with direct actions on neurons, glial cells, and the neurovascular unit (35, 49, 92).

This integrated mode of action is particularly relevant for neurological conditions characterized by metabolic vulnerability, where neurocentric therapeutic strategies have shown limited efficacy (51, 111). By simultaneously targeting metabolic and neural pathways, GLP-1RAs represent a shift toward systemic interventions for brain disorders (110–112).

## 8 Safety, pharmacokinetics, and current limitations

Despite growing interest in the neurological potential of GLP-1 receptor agonists, several safety considerations, pharmacokinetic uncertainties, and evidence gaps must be addressed before widespread neurological application (26, 113).

### 8.1 Central nervous system safety

GLP-1RAs have an established safety profile in metabolic disease (50, 114). From a neurological standpoint, available clinical data do not indicate overt neurotoxicity or increased risk of major neurological adverse events (115).

However, most safety data derive from populations without primary neurological disorders, and central nervous system outcomes have rarely been predefined endpoints (37). Gastrointestinal adverse effects may indirectly affect neurological patients with frailty or autonomic dysfunction (116). Moreover, the long-term consequences of sustained central GLP-1 receptor activation on neuronal excitability and synaptic remodeling remain incompletely characterized (5, 22).

Accordingly, dedicated neurocentric safety assessments are warranted, particularly in populations with neurodegenerative or cerebrovascular disease (37, 117).

### 8.2 Pharmacokinetics and brain exposure

The extent and mechanisms by which GLP-1RAs access the brain remain incompletely understood (25). Most GLP-1RAs are large peptide molecules with limited passive diffusion across the blood–brain barrier, raising uncertainty regarding direct central bioavailability (24, 118).

Experimental data suggest that central effects may arise through complementary mechanisms, including limited blood–brain barrier transport, activation of receptors on cerebral endothelial cells, and indirect signaling via vagal afferents or circumventricular organs (17, 119). Quantitative data on regional brain exposure, receptor occupancy, and pharmacodynamics, however, remain scarce (25, 120).

This uncertainty complicates interpretation of preclinical findings and highlights the need for advanced approaches to assess central drug distribution, including molecular imaging and cerebrospinal fluid biomarkers (121, 122).

### 8.3 Limitations of current evidence

The current evidence base is characterized by several limitations. Mechanistic insights largely derive from preclinical models that may not capture human disease heterogeneity, comorbidities, treatment timing, or longitudinal progression (123). In addition, clinical studies evaluating neurological outcomes are often secondary analyses of trials designed for metabolic or cardiovascular endpoints, resulting in limited endpoint sensitivity, incomplete neurological phenotyping, and insufficient power for disease-specific conclusions (124, 125).

Heterogeneity among GLP-1RAs in molecular structure, receptor affinity, half-life, BBB penetration, CNS exposure, and dosing interval further complicates interpretation of class effects (118, 126). Consequently, biological and clinical effects may be agent-specific rather than uniformly class-associated. Trial design must therefore consider the selected molecule, dose, route, treatment duration, disease stage, and biological target, rather than assuming interchangeability across GLP-1RAs (127).

A further limitation is endpoint sensitivity. Many neurological trials rely on clinical scales that may be insensitive to slow biological effects, while biomarkers of neurodegeneration, neuroinflammation, and neurovascular injury are not consistently incorporated (117, 125, 128). These limitations underscore the need for rigorously designed neurological trials with prespecified endpoints, adequate follow-up, biomarker enrichment, and transparent separation of primary, secondary, and *post hoc* analyses.

### 8.4 Trial design, endpoint sensitivity, and bias

Neurological trials of GLP-1RAs face several recurrent design challenges. First, disease stage may strongly influence treatment responsiveness: metabolic or neurovascular modulation may be more detectable before advanced neuronal loss, whereas late-stage disease may be less reversible. Second, short follow-up and conventional clinical scales may fail to detect gradual biological effects. Third, *post hoc* analyses of cardiovascular or metabolic trials can be informative, but they are vulnerable to endpoint multiplicity, selection bias, and incomplete neurological phenotyping.

Future trials should prespecify neurological endpoints, define biomarker-based inclusion criteria where appropriate, distinguish symptomatic improvement from disease modification, and report negative or neutral findings with the same detail as positive outcomes. These requirements are especially important for AD, PD, and stroke, where preclinical consistency has not always translated into clinical efficacy.

## 9 Future perspectives and potential breakthroughs

### 9.1 Multi-receptor agonists

Dual and triple incretin receptor agonists, including GLP-1/GIP and GLP-1/GIP/glucagon receptor agonists, represent a major pharmacological advance (129, 130). These agents may exert broader central effects by engaging complementary signaling pathways involved in energy homeostasis, inflammation, and neurovascular regulation (131, 132).

Whether multi-receptor agonism confers superior neurological benefit through enhanced systemic metabolic control, direct central actions, or synergistic modulation of the neurovascular unit remains to be determined (118).

### 9.2 Combination therapeutic strategies

Given the multifactorial nature of neurological disease, GLP-1RAs may be particularly suited to combination approaches (133). Acting as metabolic and vascular stabilizers, they may enhance the efficacy of neuroprotective or disease-modifying agents targeting complementary pathways (128).

Such strategies align with an emerging view of neurological disease as a systemic disorder with central manifestations rather than an isolated brain pathology (134).

### 9.3 Neuroimaging and biomarker integration

A major barrier to translation is the lack of sensitive endpoints capturing early neuroprotective effects (135). Advanced neuroimaging techniques and fluid biomarkers offer opportunities to interrogate neurovascular function, neuroinflammation, and network-level brain integrity (121, 136).

Integration of imaging and molecular biomarkers may help distinguish direct central effects of GLP-1RAs from secondary metabolic benefits, strengthening causal inference in clinical trials (137).

### 9.4 Redefining clinical trial endpoints

Traditional clinical scales may be insufficiently sensitive to detect gradual, pleiotropic effects of GLP-1RAs (138). Composite endpoints integrating cognitive measures, imaging markers, and molecular biomarkers may better reflect the biological targets of GLP-1–based therapies and accelerate identification of disease-modifying signals (139, 140).

## 10 Conclusion

GLP-1 receptor agonists have attracted increasing interest in neurology because they act on metabolic, inflammatory, mitochondrial, and neurovascular pathways that are relevant to several brain disorders. Current evidence supports further investigation of these agents in neurodegenerative, cerebrovascular, and metabolism-related neurological conditions.

The available evidence is strongest for biological rationale and vascular risk reduction, whereas disease-specific neurological efficacy remains uncertain. PD studies provide encouraging but biomarker-incomplete signals; AD trials have been mixed or negative; and stroke evidence currently supports prevention more strongly than acute treatment. These differences should be considered when interpreting preclinical and clinical findings.

Future progress will depend on rigorously designed neurological trials, biomarker-informed endpoints, careful agent selection, and explicit analysis of disease stage and metabolic phenotype. If these issues are addressed, GLP-1RAs may help define new treatment strategies for neurological diseases in which metabolic and vascular mechanisms contribute to disease onset or progression.
